# On the desmitracheate “micronetine” genus *Nippononeta* Eskov, 1992 (Araneae, Linyphiidae)

**DOI:** 10.3897/zookeys.484.8663

**Published:** 2015-03-09

**Authors:** Ming Yan, Xiaokai Liang, Lihong Tu

**Affiliations:** 1 College of Life Sciences, Capital Normal University, Xisanhuanbeilu Str. 105, Haidian Dist. Beijing, 100048, P. R. China

**Keywords:** Taxonomy, desmitracheate, scaped epigynum, genital morphology, “micronetine”

## Abstract

The desmitracheate system in a “micronetine” genus *Nippononeta* Eskov, 1992 is recognized for the first time in the present study. This makes the subfamilial placement of this genus problematic. A morphological study was conducted for *Nippononeta kurilensis* Eskov, 1992 (the type species of *Nippononeta*) and *Nippononeta coreana* (Paik, 1991). Characters of genitalia and tracheal system, as well as some somatic characters were studied in detail by using scanning electronic microscopy (SEM), and compared with those of *Agyneta*. Updated descriptions of the genus *Nippononeta* and its two species are presented. Putative synapomorphies for *Nippononeta* and *Agyneta* are provided, as well as some putative synapomorphies shared by the two genera. The results imply that both scaped epigynum and desmitracheate tracheal system are probably homoplastic. The placement of *Nippononeta* and *Agyneta* within Linyphiidae need to be resolved in future studies.

## Introduction

Linyphiidae Blackwall, 1859 is the second largest spider family, including over 4,500 species (World Spider Catalog 2014). The genital characters are species-specific in linyphiids, and provide rich information for species identification, taxonomy, and phylogenetic reconstruction (e.g. Hormiga 2000; Miller and Hormiga 2004; Hormiga and Scharff 2005; Tu and Hormiga 2011). Seven linyphiid subfamilies are recognized (Tanasevitch 2014); however, the results of phylogenetic analysis based on molecular data show that most of them are not monophyletic (Arnedo et al. 2009).

Classical taxonomy of Linyphiidae is often confusing because of the characters overlapping among groups. The currently accepted seven subfamilies are based on different characters. For example, the epigynum furnished with a scape carrying copulatory grooves and openings (referred to as “scaped epigynum” below) was used as the main diagnostic feature for Micronetinae Hull, 1920 (Millidge 1984; Saaristo and Tanasevitch 1996), and the desmitracheate tracheal system, in which the median trunks are extensively branched and extend into the prosoma, was used as the main diagnostic feature for Erigoninae (Blest 1976; Millidge 1984). However, both scaped epigynum and desmitracheate system can be also found in the “micronetine” genera: *Tennesseellum* Petrunkevitch, 1925, *Agyneta* Hull, 1911 (including *Meioneta* Hull, 1920, now a junior synonym of *Agyneta*), and *Anibontes* Chamberlin 1924 (Millidge 1986; Hormiga 1994; Dupérré 2013). At the same time, neither of these two characters is found in some erigonines: *Asthenargus* Simon & Fage, 1922, *Gongylidiellum* Simon, 1884, and *Ostearius* Hull, 1911 (Hormiga 2000; Miller and Hormiga 2004). This makes the subfamilial placement of abovelisted genera problematic. A recent phylogenetic study of Linyphiidae based on molecular and morphological data (Arnedo et al. 2009) indicated that the “micronetines”, as a paraphyletic group, together with the erigonines form the “micronetines+erigonines” clade. The morphology data suggested a single origin of the desmitracheate system, as a synapomorphy for Erigoninae, while the total evidence favored double origins.

Micronetinae is a large subfamily, which currently includes 1212 species placed in 91 genera (Tanasevitch 2014). After it was redefined by Saaristo and Tanasevitch (1996), Micronetinae has been extensively revised at genus level based on genital characters (e.g. Saaristo and Tanasevitch 2002a, b; Saaristo and Marusik 2004; Saaristo et al. 2006; Tu et al. 2006; Tu and Li 2006; Dupérré 2013; Sun et al. 2014). Comparative studies show that a series of transitions exist among the forms of scaped epigynum (Tu and Hormiga 2010). Five characters were proposed to accommodate interspecific variation in tracheal anatomy (Miller and Hormiga 2004; Arnedo et al. 2009). These imply a more complex pattern of the relationships among “micronetines” and erigonines than previously suggested. Accordingly, analyzing more groups that possess transitional characters between the typical “micronetine” and erigonine versions may help us infer the character evolution and resolve the phylogenetic relationships among linyphiid groups.

In the present study, we report another “micronetine” genus *Nippononeta* Eskov, 1992, having a scaped epigynum, but also a desmitracheate system. The genus *Nippononeta* was separated from *Agyneta*, the senior synonym of *Meioneta* (Eskov, 1992). We conducted a morphological study of *Nippononeta*. Characters of the genitalia and tracheal system, as well as some somatic characters, were documented with SEM images for *Nippononeta kurilensis* Eskov, 1992 (the type species of *Nippononeta*) and *Nippononeta coreana* (Paik, 1991), and compared with those of *Agyneta*, as well as those of Erigoninae. Putative synapomorphies were proposed for *Nippononeta* and *Agyneta*, which need to be tested in future studies.

## Materials and methods

Specimens were examined and measured using a Leica M205A stereomicroscope. Male palps and epigyna were examined after they were dissected from the body. Left structures (e.g. palps, legs, etc.) were depicted. Embolic divisions were excised by breaking the membranous column which connects the suprategulum and the radix. Male palps and epigyna were cleared in methyl salicylate. Scanning electron microscopy (SEM) images were taken under a Hitachi S-3400N scanning electron microscope at the China Agricultural University. For SEM examination, the specimens were prepared following Álvarez-Padilla and Hormiga (2008). SEM images of the embolic division taken from the right palp were mirrored to match those taken from the left palp. All examined specimens are deposited in the College of Life Sciences, Capital Normal University, China (CNU). Terminology of genital and somatic characters follows Tu and Hormiga (2010) and Hormiga (2000) respectively. Anatomical abbreviations used in the text and figures are:

### Somatic morphology

AC aciniform gland spigot(s)

AG aggregate gland spigot(s)

ALS anterior lateral spinneret

CY cylindrical gland spigot(s)

FL flagelliform gland spigot

MAP major ampullate gland spigot

mAP minor ampullate gland spigot

PI piriform gland spigot(s)

PLS posterior lateral spinneret

PMS posterior median spinneret

Male palp

ARP anterior radical process

AX apex of embolus

CRL cymbial retrolateral lobe

DTA distal tibial apophysis

E embolus

EBT embolus basal tooth(teeth)

EC embolus column

EM embolic membrane

EP embolus proper

LC lamella characteristica

P paracymbium

PF proximal cymbial fold

PH pit hook

PHS pit hook sclerite

PTP proximal tibial process

R radix

RTP retrolateral tibial process

SPT suprategulum

T tegulum

TA terminal apophysis

TH thumb of embolus

Epigynum

CG copulatory groove

DP dorsal plate

LL lateral lobe on sacpe

SC scape

ST stretcher

TDF transversal dorsal fold of epigynum

TP turning point of copulatory groove

VP ventral plate

## Taxonomy

### Linyphiidae Blackwall, 1859

#### Nippononeta

Taxon classificationAnimaliaAraneaeLinyphiidae

Eskov, 1992

##### Composition.

The genus includes 24 species; the type species is *Nippononeta kurilensis* Eskov, 1992.

##### Diagnosis (updated).

**Figure 1.**
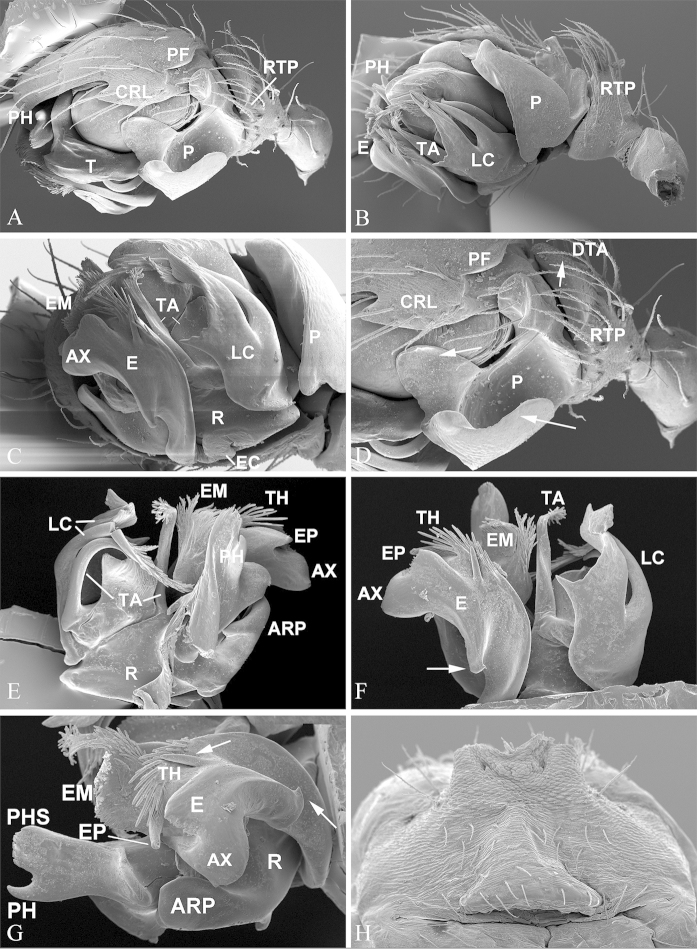
*Nippononeta kurilensis*. **A–G** male palp **A** retrolateral **B** ventral **C** anteroventral **D** detail of A, arrows indicate the serrated surface of DTA (upper), median branch of paracymbium (left) and outer margin fold continue with distal arm (lower) **E–G** embolic division **E** dorsal **F** ventral, arrow indicates basal hook of embolus **G** embolus, ventral, upper arrow indicates the last strongest spine of thumb; lower arrow indicates basal hook of embolus **H** anterior part of male abdomen, ventral, shows epiandrous gland spigots absent. ARP anterior radical process; AX apex of embolus; CRL cymbial retrolateral lobe; DTA distal tibial apophysis; E embolus; EC embolus column; EM embolic membrane; EP embolus proper; LC lamella characteristica; P paracymbium; PF proximal cymbial fold; PH pit hook; PHS pit hook sclerite; R radix; RTP retrolateral tibial process; T tegulum; TA terminal apophysis; TH thumb of embolus.

**Figure 2.**
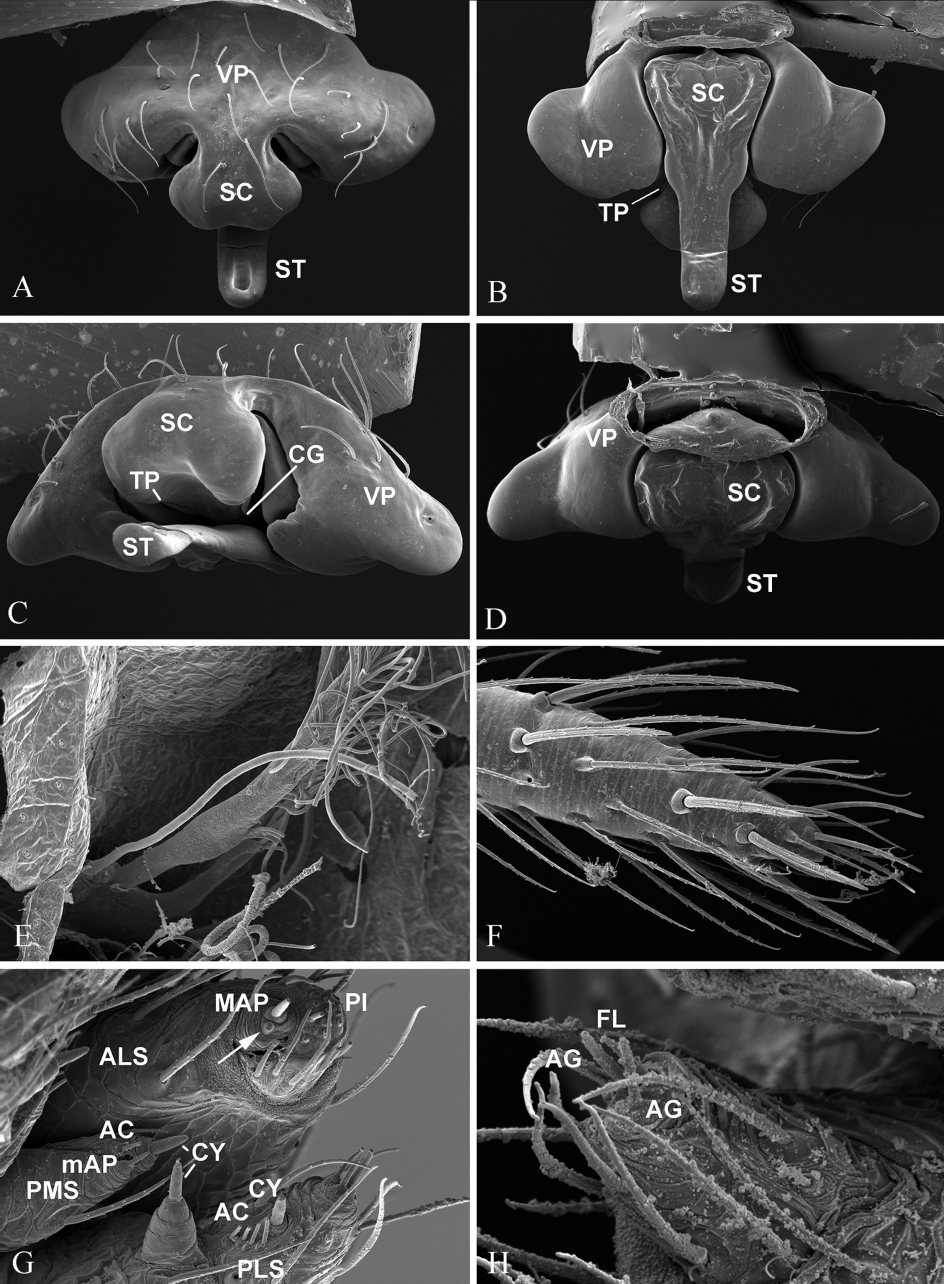
*Nippononeta kurilensis*. **A–D** epigynum. **A** ventral **B** dorsal **C** caudal **D** anterior **E** tracheal system, with soft tissue digested **F** female palp **G** female spinneret spigots, arrow indicates MPA nubbin on ALS **H** male PLS spigots. AC aciniform gland spigots; AG aggregate gland spigots; ALS anterior lateral spinneret; CG copulatory groove; CY cylindrical gland spigots; FL flagelliform gland spigot; MAP major ampullate gland spigot; mAP minor ampullate gland spigot; PI piriform gland spigots; PLS posterior lateral spinneret; PMS posterior median spinneret; SC scape; ST stretcher; TP turning point; VP ventral plate.

*Nippononeta* species are similar to *Agyneta* in many genital characters and the desmitracheate system, but differ in the presence of a dorsal pattern on the abdomen, which is absent in most *Agyneta*. Male palps of *Nippononeta* can be distinguished from *Agyneta* by the conical elevation on the cymbium absent in the former (Fig. 1A), present in the latter; the presence of proximal cymbial fold (Fig. 1D) and the spine-like embolus thumb (Fig. 1G) in *Nippononeta*, absent in *Agyneta*. The scaped epigynum in *Nippononeta* can be distinguished by its narrowed epigynal basal part covered by a transversal fold, the well developed stretcher and remnant lateral lobes (Fig. 2A–B), while in *Agyneta* the epigynal basal part is normal, the stretcher usually small or absent, but the lateral lobes are well developed bearing lateral pockets and copulatory openings (Tu and Hormiga 2010: fig. 6a).

##### Description (updated).

**Figure 3.**
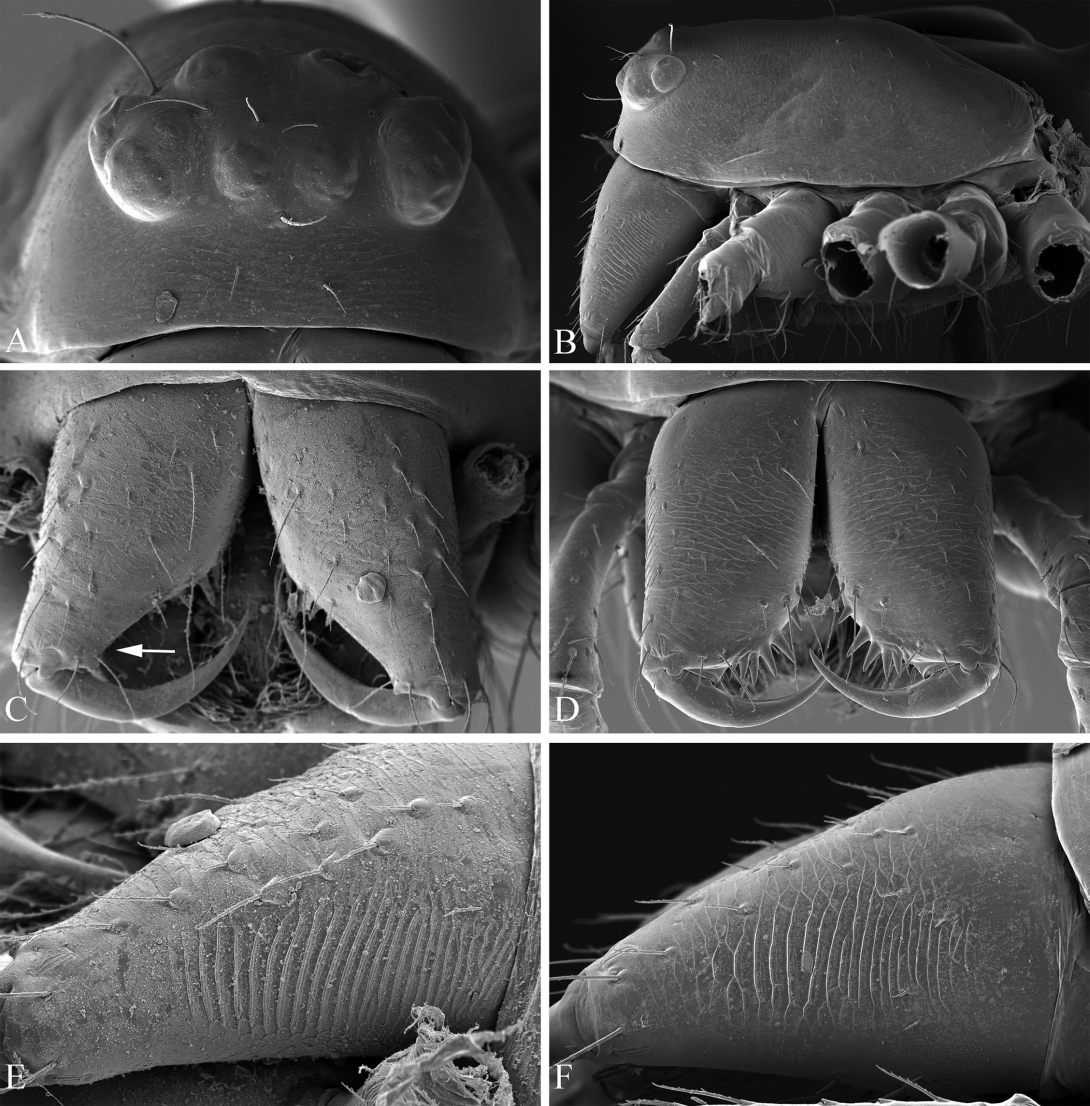
*Nippononeta kurilensis*. **A–B** female prosoma. **A** frontal **B** lateral (**C–F**) chelicera **C** male, frontal, arrow indicates narrowed fang base **D** female, frontal **E** male, ectal **F** female, ectal.

Chelicerae of normal size, with narrowed fang base and stronger stridulatory ridges in the male than in the female (Fig. 3C–F). Female palpal claw absent (Fig. 2F). Tracheal system: Median trunks wider in diameter than lateral pair, highly branched and extending into prosoma (Fig. 2E); tracheoles with taenidia. Lateral pair and median trunks arising independently from spiracular atrium. Epiandrous gland spigots absent in the male (Fig. 1H). Spinnerets: PLS in females having the mesal cylindrical gland spigot base enlarged (Fig. 2G), the triplet formed by one flagelliform and two aggregate gland spigots present in males PLS (Fig. 2H). For other somatic characters and measurements see Eskov (1992).

*Male palp* (Fig. 1A–G). Tibia short, with serrated distal apophysis and pointed retrolateral process, sometimes with additional proximal process. Cymbium with small retrolateral lobe and proximal fold above paracymbial base. Paracymbium U-shaped, with median branch arising from inner margin. Distal suprategular apophysis modified into pit hook and hook sclerite. Embolic membrane large, with many papillae. Embolic division: radix boat shaped, connected to embolus by S-shaped membranous column. Embolus with pointed proper and serrated area, thumb modified into many spine-like projections, large apex and basal apophysis. Lamella characteristica usually splitting into two or three branches, at least one of them ribbon like with thread projections distally. Terminal apophysis composed by one large basal sclerite and one or two branches with papillae apex.

*Epigynum* (Figs 2A–D, 5). Epigynal basal part narrowed, covered by transversal fold formed by the tegument connecting to dorsal side of epigynum. Median plate absent and epigynal cavity dorsally opened. Scape sigmoid folded with well developed stretcher furnished with a pit; lateral lobes remnant; copulatory openings covered by folded scape.

##### Distribution.

China, Japan, Korea, Russia.

#### Nippononeta kurilensis

Taxon classificationAnimaliaAraneaeLinyphiidae

Eskov, 1992

Nippononeta kurilensis Eskov, 1992: 159, f. 27–30 (Dmf); Ono et al. 2009: 318, f. 899–902 (mf).

##### Material examined.

1♂ and 1♀ (CNU), Russia, Sakhalin Island, near Novoalexandrovsk, 11 Sept. 1992, A. Basarukin leg.

##### Diagnosis.

**Figure 4.**
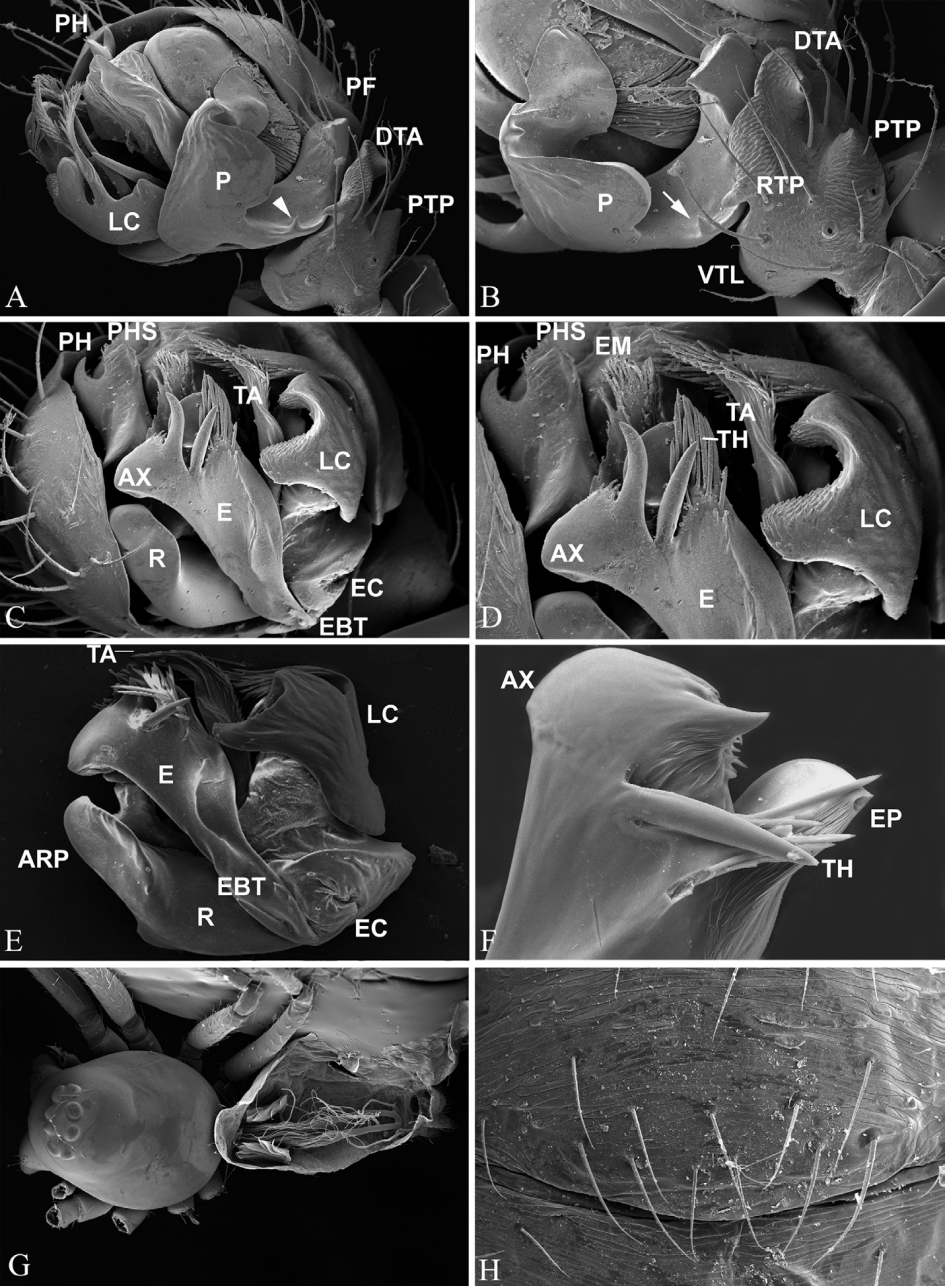
*Nippononeta coreana*. **A–F** male palp. **A** retrolateral, arrow indicates outer margin tooth **B** detail of A, arrow indicates outer margin tooth **C** ventral **D** detail of C **E** embolic division, ventral **F** embolus, dorsal **G** male body with soft tissues digested, shows tracheal system **H** anterior part of male abdomen, ventral, shows epiandrous gland spigots absent. ARP anterior radical process; AX apex of embolus; DTA distal tibial apophysis; E embolus; EBT embolus basal teeth; EC embolus column; EM embolic membrane; EP embolus proper; LC lamella characteristica; P paracymbium; PF proximal cymbial fold; PH pit hook; PHS pit hook sclerite; PTP proximal tibial process; R radix; RTP retrolateral tibial process; TA terminal apophysis; TH thumb of embolus.

**Figure 5.**
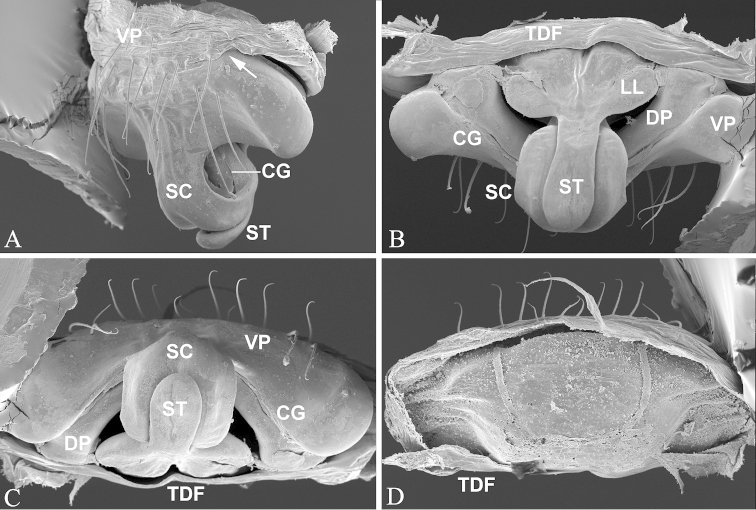
*Nippononeta coreana*, epigynum. **A** ventrolateral **B** dorsal **C** caudal **D** anterior, with soft tissues digested. CG copulatory groove; DP dorsal plate; LL lateral lobe on sacpe; SC scape; ST stretcher; TDF transversal dorsal fold of epigynum; VP ventral plate.

The male of *Nippononeta kurilensis* is distinguished from *Nippononeta coreana* and all other *Nippononeta* species by: 1) the absence of proximal tibial process (Fig. 1A, D), present in the latter (Fig. 4A–B); 2) the outer margin fold of the U-shaped paracymbium is continued with the distal arm in *Nippononeta kurilensis* (Fig. 1A, D), only a small pointed tooth in *Nippononeta coreana* (Fig. 4A–B); 3) the anterior sclerites of lamella characteristica with smooth margin in *Nippononeta kurilensis* (Fig. 1F), while that in *Nippononeta coreana* serrated (Fig. 4D), and 4) the single embolus basal tooth hook-shaped in *Nippononeta kurilensis* (Fig. 1F), whereas in *Nippononeta coreana* the embolus has three basal teeth, one of them spine-like (Fig. 4E). The female can be distinguished by the appearance of epigynum: diamond shaped in *Nippononeta kurilensis* (Fig. 2A), T-shaped in *Nippononeta coreana* (Fig. 5).

##### Description.

Somatic and genital characters as in the genus description.

##### Distribution.

Russia (Sakhalin, South Kuril Islands) and Japan (Hokkaido).

#### Nippononeta coreana

Taxon classificationAnimaliaAraneaeLinyphiidae

(Paik, 1991)

Macrargus coreanus Paik, 1991: 2, f. 30-38 (Df).
Nippononeta coreana : Eskov 1992: 159 (Tf from *Macrargus*); Li et al. 1996: 10, f. 1–7 (f, Dm); Song et al. 1999: 199, f. 113A–C (mf); Yin et al. 2012: 539, f. 256a–e (mf).

##### Material examined.

2♂ and 2♀ (CNU), China, Sichuan Province, Tianquan County, Mt. Erlangshan Natural Forest Park, 8 July 2004, L. Tu leg.

##### Diagnosis.

See the diagnosis of *Nippononeta kurilensis*.

##### Description.

Other genital characters see the description by Paik (1991).

**Figure 6.**
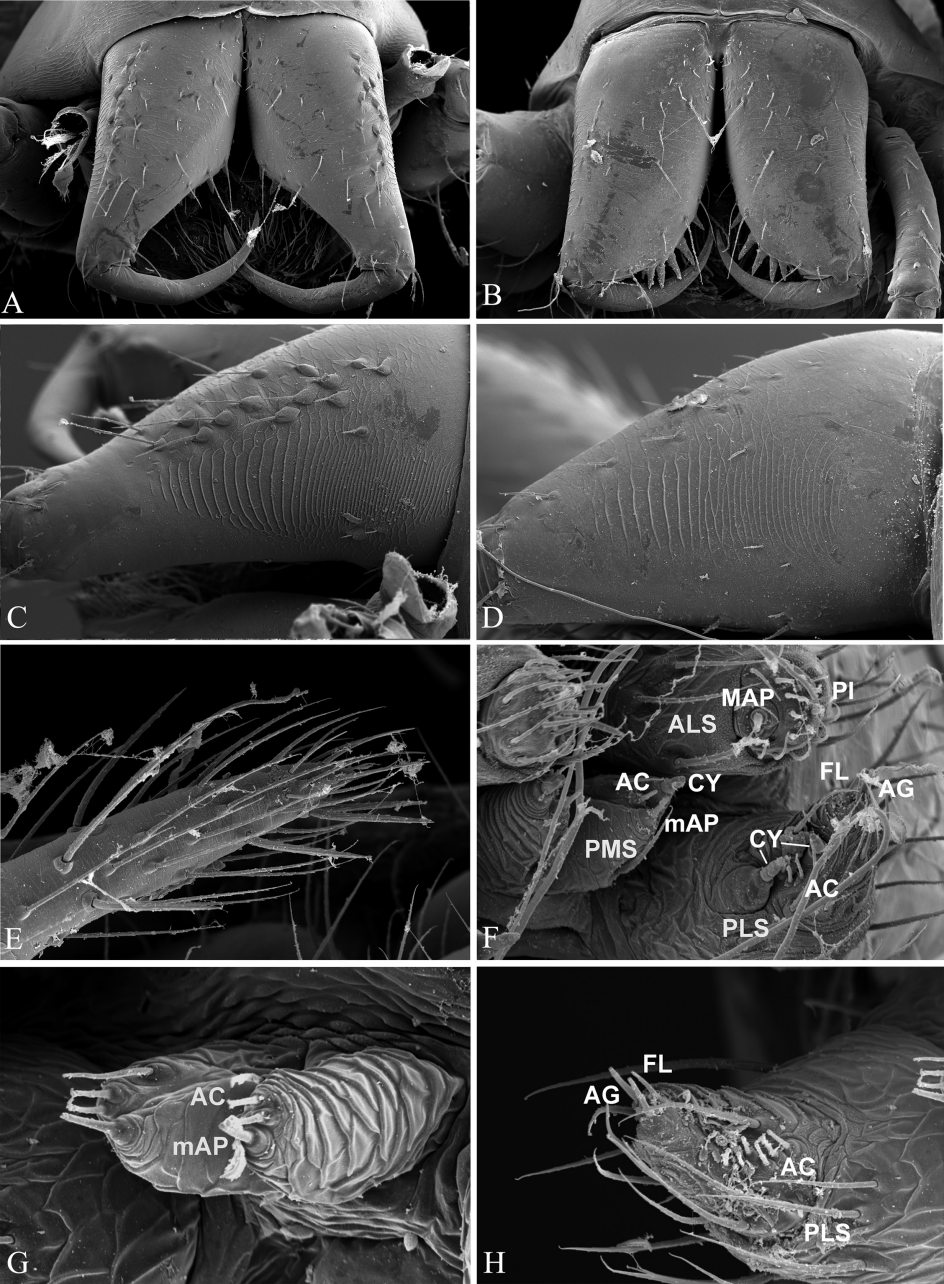
*Nippononeta coreana*. **A–D** chelicerae **A** male, front **B** female, front **C** male, ectal **D** female, ectal **E** female palp **F–G** spinnerets **F** female **G** male, PMS spigots **H** male PLS spigots. AC aciniform gland spigots; AG aggregate gland spigots; ALS anterior lateral spinneret; CY cylindrical gland spigots; FL flagelliform gland spigot; MAP major ampullate gland spigot; mAP minor ampullate gland spigot; PI piriform gland spigots; PLS posterior lateral spinneret; PMS posterior median spinneret.

##### Distribution.

China (Guangxi, Hunan, Hubei, Jilin, Sichuan), Korea.

## Discussion

The putative synapomorphies based on genital characters suggest that the four desmitracheate “micronetine” genera: *Nippononeta*, *Agyneta*, *Tennesseellum*, and *Anibontes* have a common ancestor. Eskov (1992) erected the genus *Nippononeta* to allocate those species which were not consistent with several generic diagnoses of *Meioneta* (a junior synonym of *Agyneta*). Although *Nippononeta* and *Agyneta* have a desmitracheate system, the genital characters of both genera are of a typical “micronetine” type: male palpal embolic division has well developed lamella characteristica and terminal apophysis, and females have a scaped epigynum. At the same time, in most Erigoninae, lamella characteristica and terminal apophysis are secondarily lost (Hormiga 2000), and a scaped epigynum has never been documented. There are several putative synapomorphies based on genital anatomy in both genera. For *Nippononeta*, putative synapomorphies include: the embolus thumb modified into many spine-like projections (Fig. 1G), the presence of proximal cymbial fold (Fig. 1A), and the narrowed epigynal basal part covered by the transversal dorsal fold (Fig. 2B). For *Agyneta*, putative synapomorphies include: the presence of conical cymbial elevation, and the scaped epigynum with the stretcher remnant or absent, but a pair of well developed lateral lobes bearing lateral pockets and copulatory openings (Tu and Hormiga 2010: fig. 6a). Further, putative synapomorphies for (*Nippononeta* + *Agyneta*) clade are: the serrated distal tibial apophysis (Fig. 1D) and the absence of median plate (Fig. 2B). According to Dupérré (2013), the genitalia of *Tennesseellum* and *Anibontes* have the exact same configuration as that of *Agyneta*, only different in small details. Therefore, based on genital synapomorphies, *Nippononeta* should form the sister clade to the group including the other three “micronetine” genera (*Agyneta*, *Tennesseellum*, and *Anibontes*).

The results of phylogenetic analyses support the single origin of desmitracheate system (Arnedo et al. 2009; Hormiga 2000; Miller and Hormiga 2004). Four types of tracheal anatomy have been recognized in Linyphiidae: desmitracheate, haplotracheate, and two intermediate types (Miller and Hormiga 2004). Based on the examination for 121 linyphiids belonging to 98 genera, Blest (1976) found the desmitracheate system in 85 erigonines belonging to 65 genera. This tracheal system is addressed as an erigonine type, although there are eight erigonine species with the simple (haplotracheate) type. At the same time, 19 “micronetine” species belonging to 16 genera, and all other linyphiids examined by Blest (1976) had a haplotracheate system. Later studies found that the typical “micronetine” genera *Agyneta*, *Tennesseellum*, *Anibontes* (Millidge 1986; Hormiga 1994; Dupérré 2013) and *Nippononeta* also have a desmitracheate system. Although a phylogenetic analysis of linyphiids rendered “micronetines” paraphyletic, the morphological partition suggests that the desmitracheate system in linyphiids has a single origin (Arnedo et al. 2009), with secondary reduction to the haplotracheate system within erigonines (Miller and Hormiga 2004).

Comparative studies on the tracheal morphology suggest that the transformation between haplotracheate and desmitracheate systems is not a result of a single morphological change. A total of five characters were proposed to allocate interspecific variation of tracheal anatomy in Linyphiidae (Miller and Hormiga 2004; Arnedo et al. 2009). Further, variation of the desmitracheate system was documented: the median trunk tracheoles can either have taenidia or not; the lateral tracheae can arise either independently from the spiracular atrium (Figs 2E, 4G, see also Millidge 1986: fig. 12) or from the basal part of the median trunk (Hormiga 1994: fig. 18; Dupérré 2013: figs 31–33); and the lateral tracheae can either be branching or non-branching (Millidge 1986: figs 1, 12; Hormiga 1994: fig. 18; Dupérré 2013: figs 31–33). The presence of taenidia in *Nippononeta* and *Agyneta* makes their tracheal characters similar to those observed in deeper clades within erigonines, e.g. genera *Hilaira*, *Drepanotylus*, and *Leptorhoptrum* (Blest 1976: plate 1b). At the same time, it makes *Nippononeta* and *Agyneta* different from the ‘distal erigonines’ clade, which includes generas having simple type genitalia, desmitracheate system, and the tracheoles without taenidia, e.g. *Erigone*, *Oedothorax*, and *Gonatium* (Hormiga 2000, Arnedo et al. 2009). The branching lateral tracheae, reported from some distal erigonines, e.g. *Erigone* (Millidge 1986: fig. 5; Hormiga 1994: fig. 18C), can also be found in *Tennesseellum* (Millidge 1986: fig. 1; Hormiga 1994: fig. 17). Furthermore, two intermediate types are found in several distantly related groups: *Helophora* (“micronetine”), *Allomengea* (linyphiine), *Solenysa* (“ipaine”), and some erigonines (Millidge 1986; Hormiga 1994; Miller and Hormiga 2004; Tu and Hormiga 2011). Some of them represent intermediate steps of the evolution from the haplotracheate system to the desmitracheate one, while some are results of reversals (Hormiga 2000; Miller and Hormiga 2004). Accordingly, multiple pathways are possible for the evolution of desmitracheate system. These hypotheses need to be tested with denser sampling in future studies.

Furthermore, we find that, in addition to the desmitracheate system, some confirmed synapomorphies of erigonines (Hormiga 2000; Arnedo et al. 2009) such as the presence of triplet of spigots in male PLS (Fig. 2H), the absence of epiandrous gland spigots in males (Fig. 1H), and the absence of palpal claw in females (Fig. 2F) are shared not only with *Nippononeta* and *Agyneta*, but also with some haplotracheate “micronetines”, e.g. *Microneta viaria* (Arnedo et al. 2009), *Macrargus rufus*, *Maro sublestus*, *Oreonetides vaginatus* and *Ryojius* sp. (Tu, unpublished data). This is consistent with the results of phylogenetic analysis demonsrating that “micronetines” are a paraphyletic group (Arnedo et al. 2009). The placement of *Nippononeta* and *Agyneta* within Linyphiidae and their relationships with other “micronetines” and erigonines need to be resolved in future studies.

## Supplementary Material

XML Treatment for Nippononeta

XML Treatment for Nippononeta kurilensis

XML Treatment for Nippononeta coreana
